# Psychological distress of residents in Kawauchi village, Fukushima Prefecture after the accident at Fukushima Daiichi Nuclear Power Station: the Fukushima Health Management Survey

**DOI:** 10.7717/peerj.2353

**Published:** 2016-08-31

**Authors:** Koji Yoshida, Tetsuko Shinkawa, Hideko Urata, Kanami Nakashima, Makiko Orita, Kiyotaka Yasui, Atsushi Kumagai, Akira Ohtsuru, Hirooki Yabe, Masaharu Maeda, Naomi Hayashida, Takashi Kudo, Shunichi Yamashita, Noboru Takamura

**Affiliations:** 1 Department of Health Sciences, Nagasaki University Graduate School of Biomedical Sciences, Nagasaki, Japan; 2 Education Center for Disaster Medicine, Fukushima Medical University, Fukushima, Japan; 3 Department of Molecular Medicine, Atomic Bomb Disease Institute, Nagasaki University, Nagasaki, Japan; 4 Department of Global Health, Medicine and Welfare, Atomic Bomb Disease Institute, Nagasaki University, Nagasaki, Japan; 5 Department of Radiation Health Management, Fukushima Medical University, School of Medicine, Fukushima, Japan; 6 Department of Neuropsychiatry, Fukushima Medical University, School of Medicine, Fukushima, Japan; 7 Department of Disaster Psychiatry, Fukushima Medical University, School of Medicine, Fukushima, Japan; 8 Division of Promotion of Collaborative Research on Radiation and Environment Health Effects, Atomic Bomb Disease Institute, Nagasaki University, Nagasaki, Japan; 9 Department of Radioisotope Medicine, Atomic Bomb Disease Institute, Nagasaki University, Nagasaki, Japan; 10 Department of Radiation Medical Sciences, Atomic Bomb Disease Institute, Nagasaki University, Nagasaki, Japan

**Keywords:** Fukushima Daiichi Nuclear Power Station, Nuclear disaster, Posttraumatic stress disorder, Kessler Psychological Distress Scale, Mental health, Fukushima Health Management Survey

## Abstract

**Background:**

To shed light on the mental health of evacuees after the accident at Fukushima Daiichi Nuclear Power Station (FDNPS), we evaluate the results of the Fukushima Health Management Survey (FHMS) of the residents at Kawauchi village in Fukushima, which is located less than 30 km from the FDNPS.

**Methods:**

We conducted the cross-sectional study within the framework of the FHMS. Exposure values were “anorexia,” “subjective feelings about health,” “feelings about sleep satisfaction,” and “bereavement caused by the disaster,” confounding variables were “age” and “sex,” and outcome variables were “K6 points.” We collected data from the FHMS, and employed the Kessler Psychological Distress Scale (K6) and the posttraumatic stress disorder (PTSD) Checklist Stressor-Specific Version (PCL-S) to carry out the research. A total of 13 or greater was the cut-off for identifying serious mental illness using the K6 scale. The study subjects included residents (n = 542) of over 30 years of age from Kawauchi village, and data were used from the period of January 1, 2012 to October 31, 2012.

**Results:**

A total of 474 residents (87.5%) scored less than 13 points in the K6 and 68 (12.6%) scored 13 points or more. The proportion of elderly residents (over 65 years old) among people with K6 score above the cut-off was higher than that among people with K6 score below the cut-off (44.1 vs 31.0%, p < 0.05). In addition, the proportion of residents with anorexia and mental illness among people with K6 score above the cut-off was higher than among people with K6 score below the cut-off (p < 0.001 and p < 0.05, respectively). The amount of residents who scored 44 points or more in the PCL-S among people with K6 score above the cut-off was also considerably higher than among people with K6 score below the cut-off (79.4 vs 12.9%, p < 0.001). Interestingly, the proportion of residents who scored more than among people with K6 score above the cut-off and the among people with PCL-S score above the cut-off in Kawauchi was higher than in previous studies in other locations.

**Conclusions:**

These results suggest that there are severe mental health problems, such as depression and PTSD, among adults as a consequence of the accident at the FDNPS. Our study showed that residents who lived in the evacuation zone before the disaster are at high risk psychological distress. To facilitate local residents’ recovery from Fukushima, there is a need to continue providing them with physical and mental support, as well as communication regarding the health risks of radiation.

## Introduction

On 11 March 2011, the Great East Japan earthquake struck the east coast of Japan. This natural disaster caused immense damage in Japan and resulted in severe damage to the Fukushima Daiichi Nuclear Power Station (FDNPS) (United Nations Scientific Committee on the Effects of Atomic Radiation, 2013; International Atomic Energy Agency, 2015). This nuclear accident resulted in the release of large amounts of radionuclides into the environment (Nagataki et al., 2013; Nagataki & Takamura, 2014; Endo et al., 2012; Zheng et al., 2012; Katata et al., 2012). After the accident, the Japanese and Fukushima prefectural governments immediately issued instructions for the evacuation of areas within a 20-km radius of the FDNPS. Beyond that inner radius, additional areas were designated Deliberate Evacuation Areas if there was concern that the cumulative doses of radiation might reach 20 mSv per year in those areas. As a result, many residents who lived in Fukushima Prefecture evacuated inside or outside of Fukushima Prefecture due to the fear of radiation exposure (International Atomic Energy Agency, 2015). Although the exposure dose externally and internally just after the accident itself was estimated to be very low, and despite the difficulty confirming a direct link between radiation exposure effects and any of the Fukushima residents’ physical conditions (Nagataki et al., 2013; Ishikawa et al., 2015), the accident caused anxiety about the effects on residents’ health of radioactive exposure.

In previous nuclear accidents—such as Chernobyl and the Three Mile Island accidents—psychological distress was observed (Bromet & Havenaar, 2007; Bromet, 2014). Also, the report on health impact in Chernobyl 20 years after the accident by the World Health Organization (WHO) showed that mental health was the most serious public health problem resulting from that nuclear accident (WHO, 2005a; WHO, 2005b; WHO, 2006). Based on lessons learned from past nuclear disasters, the Fukushima Health Management Survey (FHMS) has been initiated in order to assess the health impact, including the mental health, on residents in Fukushima Prefecture by the Fukushima prefectural government and Fukushima Medical University (Yasumura et al., 2012; Yabe et al., 2014).

**Figure 1:**
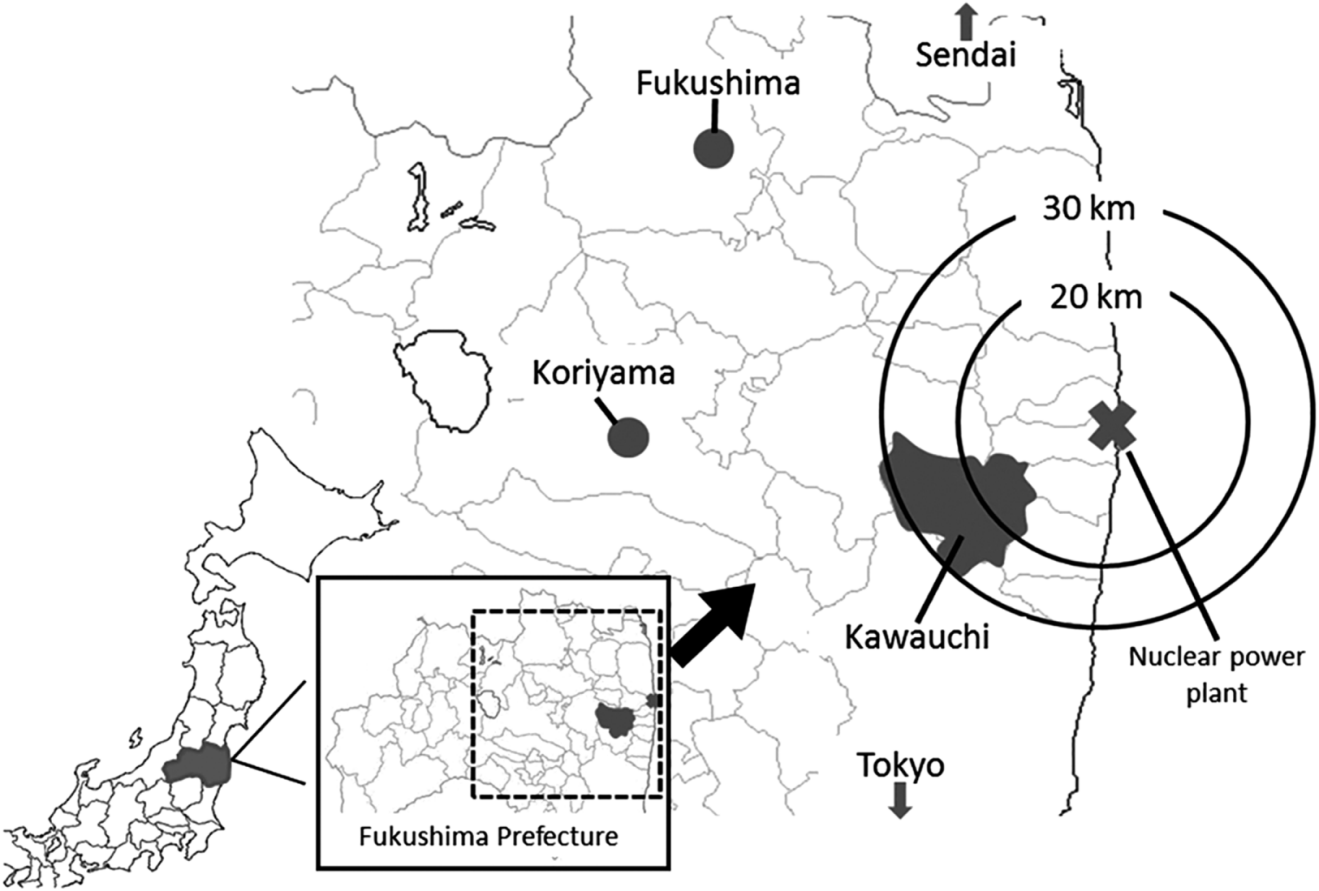
Map. Location of Kawauchi village in Fukushima Prefecture.

Kawauchi village, Fukushima Prefecture, is located within a 30 km-radius from FDNPS (Fig. 1). After the accident at FDNPS, almost all residents were evacuated outside the village. On January 31, 2012, the mayor of the village declared that residents who lived at least 20 km away from FDNPS could return to their homes because the Japanese Prime Minister had declared that the reactors had achieved a state of cold shutdown in December 2011 and that radiation doses were found to be at comparatively low levels. Kawauchi village was the first local authority whose residents returned to their hometown after the evacuation due to the accident. The number of residents returning to the village gradually increased, but around 40% of its residents remain evacuated (Kawauchi Village, 2013).

In this study, we conducted a survey of residents in Kawauchi village, in order to investigate their mental health and lifestyles after the nuclear accident of 2011.

## Materials and Methods

### Study population and data collection

This study was conducted in a cross-sectional manner using the FHMS. The aim of this survey was to monitor the long-term health and daily lives of Fukushima residents and to provide them with proper care; it was conducted via mail on about 200,000 residents who had been living in the evacuation area at the time of the disaster. The entire protocol of this survey is published elsewhere (Yabe et al., 2014). The survey includes a self-administered mental health questionnaire focusing on age, gender, disease (diabetes mellitus, hypertension, hyperlipidemia, etc.), smoking and drinking status, change of work, Kessler’s K6 (Kessler et al., 2003), posttraumatic stress disorder (PTSD) Checklist Stressor-Specific Version (PCL-S) (Blanchard et al., 1996) and others. We sent questionnaires to 2,250 residents of over 30 years of age in Kawauchi village. Of these, 1,040 (46.2%) sent responses. A total of 542 (24.1%) residents who answered all questions were included in the study analysis.

To assess the mental health status of the residents, the Japanese version of the K6 and PCL-S scales were used. The Japanese version of the K6 has been validated in previous reports (Furukawa et al., 2003; Sakurai et al., 2011). We measured non-specific mental health distress as a primary outcome using the K6. In the K6, participants were asked if they had experienced any of the following symptoms in the previous 30 days: feeling so sad that nothing could cheer them up; feeling nervous, hopeless, restless, or fidgety; feeling everything was an effort; and feeling worthless. Each question was scored on a 5-point Likert scale from zero (none of the time) to four (all of the time), with higher scores signifying worse mental health status (range: 0–24). The cut-off point for predicting severe mental illness is 13 points (Kessler et al., 2003). The PCL-S scale was used to measure traumatic symptoms, and the events we specified were the Great East Japan earthquake and the accident of FDNPS. We summed up the responses to the 17 Likert scale items in the PCL-S and classified a responder as having probable PTSD if the total score was ≥ 44 (Blanchard et al., 1996), where each question was scored from 1 to 5 (corresponding respectively to not at all, a little bit, moderately, quite a bit, or extremely. The total score range was 13–85).

Regarding risk perceptions of radiation health effects, three items were included in the questionnaire about perceptions of risk from acute, late, and hereditary effects (Lindell & Barnes, 1986). The items were rated on a 4-point Likert scale ranging from very unlikely to very likely (1, very unlikely; 2, unlikely; 3, likely; 4, very likely).

### Statistical analysis

We analyzed the residents at Kawauchi village based on each item. We also classified the residents according to K6 point range (among people with K6 score below and/or above the cut-off), and compared the proportion of each question item. We used the chi-square test and the Mann-Whitney U test to compare the data for each group. Multiple logistic regression analysis was performed to assess the effects of each variable on the K6 points adjusted for confounding variables. In this study, the dependent variable was “K6 points,” the exposure variables were “anorexia,” “subjective feelings about health,” “feelings about sleep satisfaction,” and “bereavement caused by the disaster,” and the confounding variables were “age” and “sex.” Odds ratios (ORs) and their 95% confidence intervals (95% CI) were also calculated. p values of less than 0.05 were considered to be significant. Statistical analysis was performed using SPSS Statistics 22.0 (IBM Japan, Tokyo, Japan).

### Ethics statement

This study was approved by the ethics committee of Fukushima Medical University (No. 2045) and Nagasaki University (No. 14021388) prior to its commencement. Prior to the study, we distributed the instructions about the FHMS and other surveys to residents. We considered that residents who responded to the questionnaires agreed to participate in the study.

## Results

Out of the 542 residents used for the study analysis, the number of males and females was 264 and 278, respectively, and the average age was 58.4 ± 14.9. In the survey, 474 residents scored less than 13 points in the K6 points, and 68 residents scored 13 points or more in the K6 (see Table 1). The average ages of these groups were 58.1 ± 14.8 and 60.7 ± 15.9, respectively. There was no significant difference between among people with K6 score below and above the cut-off (p = 0.16), but the proportion of elderly residents (over 65 years old) among people with K6 score above the cut-off was higher than among people with K6 score below the cut-off (44.1 vs 31.0%, p < 0.05). In addition, the proportion of residents with anorexia and mental illness among people with K6 score above the cut-off was higher than that among people with K6 score below the cut-off (p < 0.001 and p < 0.05, respectively). The proportion of residents who experienced bereavement as a result of the disaster among people with K6 score above the cut-off was higher than among people with K6 score below the cut-off (20.6 vs 9.1%, p < 0.01), and the proportion of residents with 44 points or more in the PCL-S among people with K6 score above the cut-off was considerably higher than among people with K6 score below the cut-off (79.4 vs 12.9%, p < 0.001). Conversely, the proportion of residents who expressed satisfaction with their sleep patterns and health among people with K6 score above the cut-off was lower than among people with K6 score below the cut-off (p < 0.001, respectively). There was no significant difference between the two groups regarding the rates of smokers and drinkers (p = 0.88 and p = 0.36, respectively). Also, the total number of Kawauchi residents above the K6 and the PCL-S cut-off point (n = 68 and n = 115, respectively) was greater than that of residents who have been diagnosed with mental illness (n = 28).

**Table 1:** Basic character. Residents of among people with K6 score below and/or above the cut-off in Kawauchi village.

	Among people with K6 score below the cut-off (n = 474)	Among people with K6 score above the cut-off (n = 68)	p values
	Number (%)	Number (%)	
**Sex**			
Male/Female	236/238 (49.8/50.2)	28/40 (29.4/70.6)	0.20
**Age**			
Average age	58.1 ± 14.8	60.7 ± 15.9	0.16
30–64 years old/Over 65 years old	327/147 (69.0/31.0)	38/30 (55.9/44.1)	< 0.05
**PCL**			
Less than 44 points/44 points or more	413/61 (87.1/12.9)	14/54 (20.6/79.4)	< 0.001
**Illness Yes/No**			
Mental illness	20/454 (4.2/95.8)	8/60 (11.8/88.2)	< 0.05
**Lifestyle Yes/No**			
Smoking	107/367 (22.6/77.4)	14/54 (20.6/79.4)	0.88
Drinking	217/257 (45.8/54.2)	27/41 (39.7/60.3)	0.36
Anorexia	20/454 (4.2/95.8)	16/52 (23.5/76.5)	< 0.001
Subjective feelings about health	406/68 (85.7/14.3)	27/41 (39.7/60.3)	< 0.001
Feelings about sleep satisfaction	206/268 (43.5/56.5)	5/63 (7.4/92.6)	< 0.001
**Event Yes/No**			
Bereavement caused by the disaster	43/431 (9.1/90.9)	14/54 (20.6/79.4)	< 0.01

When logistic regression analysis was conducted following adjustment for confounding factors, anorexia (OR = 2.322, p = 0.047), subjective feelings about health (OR = 0.210, p < 0.001), and feelings about sleep satisfaction (OR = 0.183, p = 0.001) were significantly associated with the K6 points (Table 2).

**Table 2:** Logistic analysis. Odds ratios and 95% confidence intervals of the study variables for among people with K6 score above the cut-off in Kawauchi village, as assessed by logistic regression analysis (n = 542).

Variable	Unit	Odds ratio	95% confidence interval	p value
**Adjusted**				
Sex	Male/Female	1.027	0.574–1.836	0.929
Age	30–64 years/Over 65 years	0.751	0.411–1.371	0.351
Anorexia	Yes/No	2.322	1.010–5.339	0.047
Subjective feelings about health	Yes/No	0.210	0.114–0.388	< 0.001
Feelings about sleep satisfaction	Yes/No	0.183	0.069–0.483	0.001
Bereavement caused by the disaster	Yes/No	0.476	0.217–1.045	0.064
**Unadjusted**				
Sex	Male/Female	0.185	0.422–1.182	0.185
Age	30–64 years/Over 65 years	0.569	0.340–0.955	0.033
Anorexia	Yes/No	6.985	3.409–14.311	0.047
Subjective feelings about health	Yes/No	0.110	0.064–0.191	< 0.001
Feelings about sleep satisfaction	Yes/No	0.103	0.041–0.261	0.001
Bereavement caused by the disaster	Yes/No	0.385	0.198–0.745	0.064

The K6 and PCL-S scores were moderately correlated (*r* = 0.77, p < 0.001), and the regression equation was H_PCL-S_ = 2*H_K6_ + 20.22. According to the regression equation, if H_K6_ is 4 points, then H_PCL-S_ is estimated to be 28 points. Even if H_K6_ is 13 points (the cut-off point for predicting severe mental illness), then H_PCL-S_ is estimated to be 46 points, which is around the cut-off point for probable PTSD.

Regarding the risk perceptions of radiation health effects in residents, 917 of 1,040 residents replied to all the questions (88.2%). Of those, 156 residents (17.0%) answered that acute radiation syndrome (ARS) had occurred due to the accident, and 407 residents (44.4%) answered that they were anxious about the health effects of radiation on children. A total of 518 residents (56.5%) answered that they were anxious about the health effects of radiation in offspring particularly.

## Discussion

Within the framework of the FHMS (Yasumura et al., 2012; Yabe et al., 2014), this mental health and lifestyle survey was conducted ten months after the disaster with the objective of providing adequate mental care and lifestyle support for evacuees at greater risk of developing mental health and lifestyle problems.

K6 was employed in this survey to estimate the general mental health of the residents. According to the results of a K6 study conducted within the FHMS framework during the same period as this study (Yabe et al., 2014), the percentage of adults who scored above the cut-off point was 14.6% (12.5% for the residents of Kawauchi). Since the previous study within the FHMS framework included residents who lived in areas near the FDNPS, there may be differing results between the two studies. In any case, these values are substantially higher than that in the usual state as reported previously (3%) (Kawakami, 2007). Also, the number of Kawauchi residents above the K6 and the PCL-S cut-off points was greater than that of residents with mental illness in our study. This result suggests that they might not receive the appropriate medical support in spite of their psychological problems. Furthermore, the ratio of residents who exceeded the cut-off of 44 for the PCL-S, reflecting a traumatic response was 21.2% in this study (21.6% in FHMS: 2011). Interestingly, our results are similar to those of previous studies targeting rescue and cleanup workers in the wake of the 9/11 World Trade Center terrorist attacks (20.1%) (Stellman et al., 2008), and to studies on lower Manhattan residents two to three years after the same attacks (15.1%) (DiGrande et al., 2008).

In addition, we compared our current results of Kawauchi village with those of Goto island in Nagasaki Prefecture, where the rate of elderly residents is similar to that of Kawaushi village (32.4 and 35.2%, respectively in 2010) (Kawauchi Village, 2013; Goto City, 2012), and found that the ratio of 13 points or more of K6 was much higher in Kawauchi residents who were 65 years or older (16.9%) than Goto residents who were 65 years or older (1%). Although socioeconomic factors should be carefully evaluated, these results suggest that mental health problems are particularly severe in Kawauchi residents probably due to the accident at the FDNPS. Also, our result from using a logistic regression analysis suggested an association between anorexia and low sleep satisfaction, and deterioration of mental health. Therefore, it is necessary to support the reduction of mental stress in evacuees, especially the elderly.

We also evaluated risk perceptions of health effects due to radiation exposure in 2012, ten months after the accident and found that 17.0% of the residents answered that they had developed ARS after the accident. Specifically, 55.4% of the residents answered that they felt health effects such as malignancies would occur later in life and 43.5% of the residents answered that they felt negative hereditary effects in offspring would arise due to radiation exposure. In the Fukushima FHMS targeted at evacuees in 2011 and 2012 (Yabe et al., 2014), 8,671 (14.4%) residents answered that they had experienced ARS after the accident; 29,000 (48.1%) felt that health effects such as malignancies would occur later in life; and 36,219 (60.2%) believed that negative hereditary effects would arise in offspring due to radiation exposure in 2011. In 2014, three years after the accident, we conducted a similar study in Kawauchi village and found that 29.8% of the residents answered that they had experienced ARS; 54.0% believed that health effects such as malignancies would occur later in life; and 49.1% believed that negative hereditary genetic effects in offspring would arise due to radiation exposure (Orita et al., 2015). These suggest that different risk perceptions concerning the health effects of radiation exposure remain in the residents of Fukushima since the initial phase of the accident.

In FHMS, the external radiation doses of residents living in the Prefecture during the accident were estimated based on their behavior during the four subsequent months (Yasumura et al., 2012). The doses were evaluated at less than 5 mSv in 99.8% of all the respondents, and even at the highest maximum dose was 25 mSv (Ishikawa et al., 2015). These results show that external radiation doses among residents in Fukushima are far below the levels that cause ARS (Bolus, 2001). After the accident, radiation health risk communication has been conducted in Fukushima and is still definitely needed for every generation in order to avoid misunderstandings about radiation exposure and its health effects (Lochard, 2007).

The present study has several limitations. First, this study was conducted only in Kawauchi, which limits the generalizability of its findings. Second, we could not obtain sufficient information on potential confounding factors such as detailed lifestyle habits. In addition, selection bias should be taken into account when interpreting the study, as the response rate was limited. Another factor to bear in mind is that, there is a report on the suitability of cut-off for K6 in Japanese, but not for PCL-S.

## Conclusions

In this study, we conducted an investigation into the psychological distress and lifestyle of evacuees ten months after the Fukushima disaster. Our findings suggested that acceptance, or a lack of such, of risk perceptions regarding the health effects of radiation exposure remained in residents since the initial phase of the accident. Our study also showed that the disaster experience and life during evacuation caused a change in people’s lifestyles and subsequent health effects, while simultaneously subjecting them to psychological distress. Furthermore, our results indicated that there is a need to provide the residents with continuous physical and mental support, as well as communication regarding radiation health risks to facilitate the recovery of Fukushima.

## Supplemental Information

10.7717/peerj.2353/supp-1 Supplemental Information 1 Dataset of the study.

## Additional Information and Declarations

### Data Deposition

The following information was supplied regarding data availability: The raw data has been supplied as Supplemental Dataset Files.
